# Abnormal glucose regulation in Chinese patients with coronary artery disease: a gender analysis

**DOI:** 10.1186/s13019-022-01848-0

**Published:** 2022-05-03

**Authors:** Juan Liu, Li-Qun He, Wei Zhu, Gang-Feng Duan, Yong Fang, Ying Feng, Li-Qun Tian, Qiong-Li Zheng

**Affiliations:** Department of Cardiology of Wuhan, No.1 Hospital, No. 215 Zhongshan Avenue, Wuhan, 430022 Hubei Province China

**Keywords:** Diabetes, Coronary artery disease, Women, IGT, OGTT

## Abstract

**Background:**

Diabetes and impaired glucose regulation are very common in patients with coronary artery disease (CAD). In this study, we aim to investigate the prevalence of abnormal glucose regulation in men and women in Chinese CAD patients.

**Methods:**

In this retrospective study, 4100 patients (male, n = 2873; female, n = 1227)with CAD were enrolled. The mean age of these patients was 63 years. The demographic data, medical history, echocardiography findings and blood investigations were collected and analyzed.

**Results:**

In this population, 953 (24%) patients had definite diagnosis of type 2 diabetes mellitus, including 636 males (23%) and 317 females (27%). There was a higher prevalence of diabetes in females than men (*p* < 0.05). For the remaining patients, 48% (n = 959) undergone an oral glucose tolerance test (OGTT), which revealed that 83 male patients (12%) and 41 female patients (16%) suffered from the type 2 diabetes (*p* > 0.05). 283 men (40%) and 105 women (41%) had impaired glucose regulation (IGR) (*p* > 0.05). Only 338 men (25%) and 109 women (19%) showed the normal glucose regulation, implying a higher prevalence of abnormal glucose regulation in females (*p* < 0.01). The odd ratio (OR) showed that women were more prone to have diabetes mellitus or IGT than men and the OR was 1.44 and 1.43 respectively.

**Conclusion:**

Abnormal glucose regulation is highly prevalent in CAD patients. The women are more prone to have diabetes mellitus or IGT than men.

## Background

Diabetes mellitus is characterized by hyperglycemia due to an absolute or relative deficit in insulin production. The chronic complication of diabetes mellitus is related to the organ damage, dysfunction, such as the retina, kidney, nervous system, heart, and blood vessels [1]. According to the International Diabetes Federation, the global diabetes prevalence in 2019 is estimated to be 9.3% (463 million people), rising to 10.2% (578 million) by 2030 and 10.9% (700 million) by 2045. So, Diabetes mellitus become a worldwide health problem and bring great challenges to global health in the twenty-first century, which will lead to a huge burden through premature morbidity and mortality [2]. A recent study showed that diabetes reached epidemic proportions in China and China will become the global centre of the diabetes epidemic. 92.4 million adults (9.7% of the adult population-10.6% men and 8.8% women) sufferred from the disease, amongst whom 60.7% are undiagnosed cases. Besides, 148.2 million adults (15.5%) have prediabetes, which confers an increased risk for overt diabetes in later life (16.1% among men and 14.9% among women) [3].

Type 2 diabetes mellitus is a well known risk factor for morbidity and mortality of coronary artery disease (CAD) and acute coronary syndrome (ACS). The presence of diabetes significantly worsens the prognosis of CAD [4–7]. In addition, impaired glucose regulation (IGR), including IGT and impaired fasting glycemia (IFG), has atherogenic potential, which explains the increased risk for cardiovascular events in these patients [8–11]. In non-diabetic individuals, the risk of CAD is higher in men than women, but diabetes associated relative risk of CAD is significantly more in female than male [12–14]. Stuides showed that risk of death from CAD associated with type 2 diabetes was about 50% greater in women than in men [15].

Therefore, knowledge about the prevalence of abnormal glucose regulation in CAD patients is of great importancefor initiating appropriate and prompt prevention, as well as therapeutic measures. In fact, many prospective and retrospective studies have been carried out [16–22], which highlighted the elevated prevalence of abnormal glucose tolerance in CAD patients. However, on the difference of abnormal glucose regulation in male and female patients is still unclear. In this study, we will compare the prevalence of diabetes mellitus and IGR in male and female Chinese CAD patients.

## Methods

### Patients and data collection

4100 patients, who were admitted in Wuhan No.1 Hospital, Wuhan, China during the period May 2008 to June 2011 were enrolled in this study. The demographic data, medical history, CAD diagnosis and treatment were collected. In addition, smoking history, blood pressure, lipid levels (total-, LDL- and HDL-cholesterol, triglycerides), blood glucose and echocardiographic findings were also collected.

### Disease and diagnostic criteria

CAD was classified as stable angina pectoris, previous myocardial infarction (MI), unstable angina pectoris, acute ST-segment elevation MI (STEMI) and non-ST-segment elevation (NSTEMI). The latter three conditions were categorized under ACS. Stable angina pectoris is a clinical entity characterized by chest discomfort, which can radiate to left arm, jaw, shoulder or back, typically induced by exertion or emotional stress and relieved by rest or nitroglycerin. Diagnosis is confirmed by presence of myocardial ischemic changes in ECG, abnormal stress tests (scintigraphy, electrocardiography or echocardiography) or > 50% stenosis of the lumen diameter in any major coronary artery seen by coronary angiography [23]. Unstable angina pectoris is described as a clinical syndrome between stable angina and acute MI with main presentations being -angina at rest, new onset angina, and increasing angina [24]. Acute MI was defined according to the European and American consensus guidelines [25]. In both STEMI and NSTEMI, there is release of detectable amount of markers (troponin I and T, creatine kinase MB) of myocyte necrosis and the terms are different with respect to the reflection of acute myocardial ischemia and necrosis in the ECG. Patients were labeled as STEMI when their ECG showed ST-segment elevation of ≥ 2 mm in two or more contiguous chest leads, ≥ 1 mm in two or more limb leads, or new left bundle-branch block, simultaneously with typical symptoms (chest pain or discomfort > 20 min duration).

Heart failure was diagnosed according to the criteria from ESC Guidelines for heart failure [26]. Grading of heart failure was performed as per the New York Heart Association criteria. Hypertension was defined as systolic blood pressure ≥ 140 mmHg, and/or diastolic blood pressure ≥ 90 mmHg, or on current antihypertensive therapy. Dyslipidaemia was defined as total cholesterol ≥ 5.20 mmol/L, and/or HDL-cholesterol ≤ 0.90 mmol/L, and/or LDL-cholesterol ≥ 3.12 mmol/L, and/or triglycerides ≥ 1.69 mmol/L, or undergoing current lipid-lowering treatment.

For definite diagnosis of type 2 diabetes, IFG and IGT were defined based on the World Health Organization classifications. IGR refers to the presence of either IFG or IGT whilst abnormal glucose regulation includes IGR and diabetes. To assess the status of glucose metabolism in the hospital setup, random blood glucose (RBG) levels were recorded on admission and fasting blood glucose (FBG) were done in the following morning. To assess the glucometabolic state of previously undiagnosed diabetes patients, standard oral glucose tolerance test (OGTT; 75 g anhydrous glucose in 250–300 ml water) was carried out. Glucose concentrations were measured according to local routines in venous plasma. The patients were classified into different groups according to Table 1, which summarizes the 2006 WHO recommendations for the diagnostic criteria for diabetes and intermediate hyperglycaemia.

**Table 1:** Classification of glucometabolic state

Glucometabolic state	Glucose concentration, mmol/L (mg/dL)	
	OGTT	FPG
Normal glucose regulation		
Fasting	< 6.1 (110)	< 6.1 (110)
2 h post-load	< 7.8 (140)	–
IGT		
Fasting	< 7.0 (126)	< 7.0 (126)
2 h post-load	7.8–11.0 (140–199)	–
IFG		
Fasting	6.1–7.0 (110–126)	6.1–7.0 (110–126)
2 h post-load	< 7.8 (140)	–
New DM (NDM)		
Fasting	≥ 7.0 (126)	≥ 7.0 (126)
2 h post-load	≥ 11.1 (126)	–

### Statistical analysis

The data were analyzed using SPSS Windows version 17. Categorical variables were presented as counts and percentages. Continuous variables were presented as median and quartiles. One-way ANOVA test, Odd Ratios (OR) and Chi-Square tests were used to assess the statistical significance of observed differences among groups.

## Results

### The basic characters for subjects

**Fig. 1:**
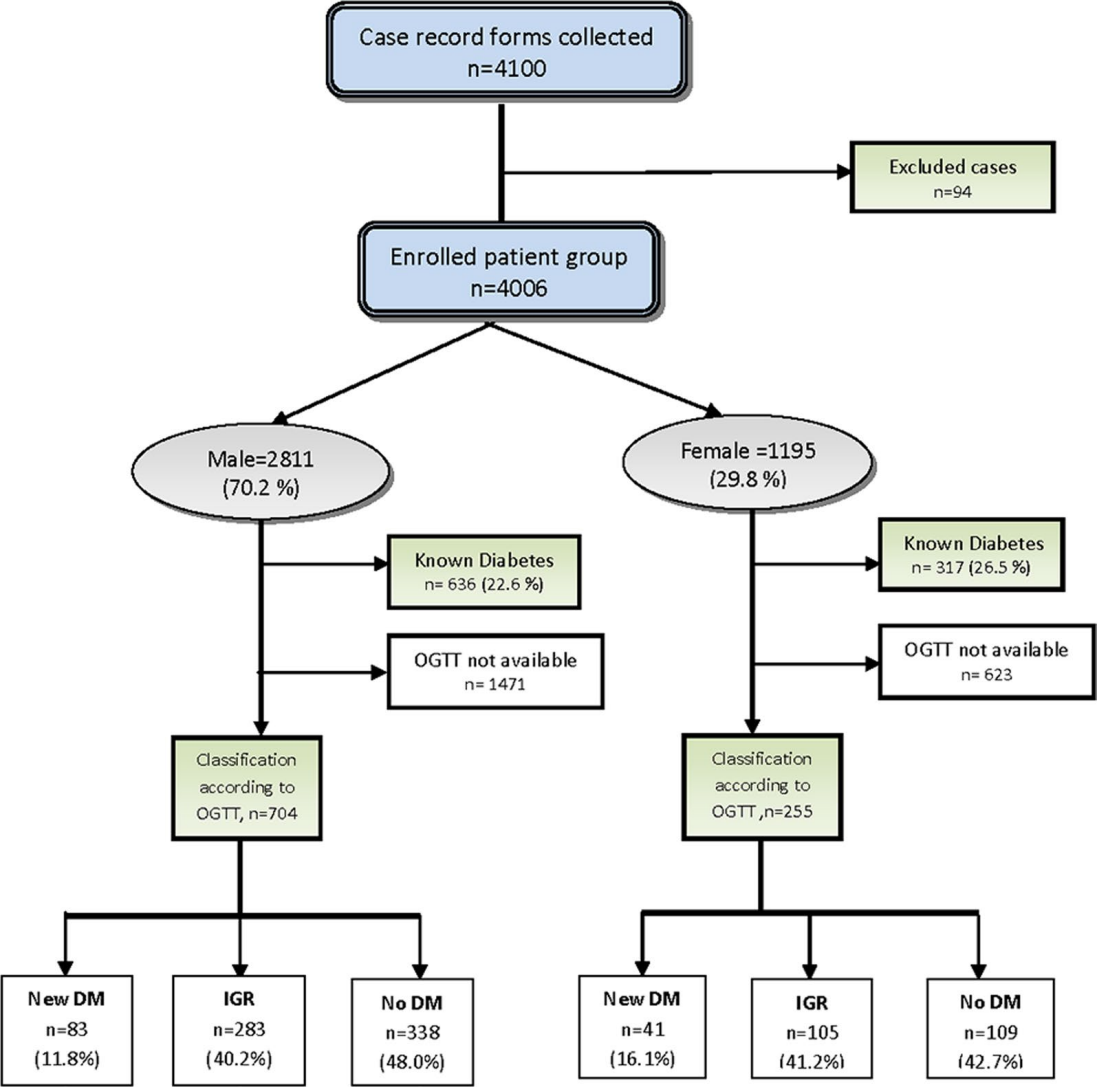
Flow diagram of the patient distribution

Out of the 4100 patients, 94 were primarily screened for diabetes rather than CAD. In this population, the male patients accounted for 70.2%) (n = 2811). The age of the patients varied from 28 to 89 years. 636 male and 317 female subjects were known diabetics. Based on the available OGTT results, the subjects were further divided into different groups according to their final diagnosis as shown in Fig. 1. The patients’ baseline demographics, including age, weight, systolic and diastolic blood pressure and heart rate were analyzed and summarized as shown in Table 2.

**Table 2:** Patient baseline demographics

Characteristics	Male (n = 1340)				Female (n = 572)			
	DM (n = 636)	NDM (n = 83)	IGR (n = 283)	Normal (n = 338)	DM (n = 317)	NDM (n = 41)	IGR (n = 105)	Normal (n = 109)
Age (years)	62 (54–70)	58 (54–67)	59 (52–69)	60 (53–69)	68 (61–74)	64 (59–70)	63 (55–68)	63 (57–68)
Weight (kg)	72 (65–78)	71 (64–80)	72 (66–78)	70 (63–75)	63 (55–70)	65 (55–80)	61 (56–69)	60 (54–67)
Systolic BP (mmHg)	130 (120–145)	130 (120–143)	130 (120–140)	130 (120–140)	140 (120–158)	142 (130–160)	130 (120–142)	130 (120–144)
Diastolic BP (mmHg)	80 (70–89)	85 (74–90)	80 (70–90)	80 (73–88)	80 (70–90)	85 (76–90)	80 (72–90)	80 (73–83)
Heart rate (beats/min)	72 (65–80)	73 (65–78)	70 (63–78)	70 (62–77)	72 (65–79)	71 (67–78)	70 (65–78)	70 (62–77)

Data were median (lower–upper quartiles)

DM, diabetes mellitus; NDM, new diabetes mellitus; IGR, impaired glucose regulation

In both male and female groups, patients’ age with diabetes was older than those without diabetes (*p* < 0.01). For diabetic patients in the two groups, females were older than males. In contrast, there were no significant differences in age (*p* > 0.05) between male and female IGR population. The systolic blood pressure was higher in diabetic women than in men (*p* < 0.01), but not in IGR patients. There was no significant difference in diastolic pressure (*p* > 0.05) Heart rate was significantly higher in diabetic patients than non-diabetic patients irrespective of sex (*p* < 0.01). However, there was no significant difference in heart rate for diabetics and IGR patients in both genders (*p* > 0.05).

### CAD diagnosis for the subjects

The different types of coronary heart disease and baseline medical history of patients were summarized in Table 3. For diabetic and IGR patients, unstable angina pectoris was the most common form of CAD, and female had a predominance. Most of the patients with NSTEMI had diabetes mellitus (female- 92% and male- 71%). Hypertension was more common in the female group, irrespective of glucometabolic state, with a prevalence of 77%. The number of male smokers exceeded the females. However, 61% of women,who were currently smoking, had diabetes mellitus as compared to only 46% for men.

**Table 3:** CAD diagnosis and baseline medical history

Type	Male					Female				
	DM (n = 636)	NDM (n = 83)	IGR (n = 283)	Normal (n = 338)	Total (n = 1340)	DM (n = 317)	NDM (n = 41)	IGR (n = 105)	Normal (n = 109)	Total (n = 572)
CAD										
SAP	20 (3)	4 (5)	9 (3)	25 (7)	58 (4)	24 (8)	2 (5)	17 (16)	15 (14)	58 (10)
Previous MI	128(20)	8 (10)	43 (15)	38 (11)	217 (16)	41 (13)	6 (15)	8 (8)	6 (6)	61 (11)
ACS										
UAP	319 (50)	39 (47)	151(53)	215 (64)	724 (54)	181(57)	24 (59)	68 (65)	81 (74)	354 (62)
STEMI	120 (19)	29 (35)	68 (24)	55 (16)	272 (20)	49 (16)	7 (17)	12 (11)	7 (6)	75 (13)
NSTEMI	49 (8)	3 (4)	12 (4)	5 (2)	69 (5)	22 (7)	2 (5)	0 (0)	0 (0)	24 (4)
Medical history										
Hypertension	461 (73)	53 (64)	172(61)	203 (60)	889 (66)	258 (81)	31 (76)	74 (71)	79 (73)	442 (77)
Stroke	12 (2)	0 (0)	5 (2)	1 (< 1)	18 (1)	7 (2)	1 (2)	0 (0)	3 (3)	11 (2)
Heart failure	117 (18)	13 (16)	43 (15)	34 (10)	207 (15)	72 (23)	8 (20)	13 (12)	16 (15)	109 (19)
Smoking										
Current	367 (58)	54 (65)	182 (64)	195 (58)	798 (60)	19 (6)	5 (12)	4 (4)	3 (3)	31 (5)
Former	43 (7)	3 (4)	14 (5)	24 (7)	84 (6)	2 (1)	0 (0)	1 (1)	0 (0)	3 (1)
Never	226 (35)	26 (31)	87 (31)	119 (35)	458 (34)	296 (93)	36 (88)	100 (95)	106 (97)	538 (94)

Data are number of patients (%)

SAP, stable angina pectoris; UAP, unstable angina pectoris; DM, diabetes mellitus; NDM, new diabetes mellitus; IGR, impaired glucose regulation

### Pharmacological treatment

Drugs administered to the patients were shown in Table 4. There was a slight difference in the proportion of drugs prescribed to men and women. Aspirin was the most frequent drug to be used (97% in men and 92% in women), while diuretics were less common. β-blocker, ACEI/ARB, Statins and anti-platelet drugs were more commonly used in males (in both DM and IGR groups),whereas diuretics and CCB were more common in females. Of note, diuretics were more extensively administered to DM and IGR patients compared to those without diabetes in males, but this was different in females, where the proportion of patients receiving this drug was much less in IGR patients.

**Table 4:** Pharmacological treatment given to patients

Type	Male					Female				
	DM (n = 636)	NDM (n = 83)	IGR (n = 283)	Normal (n = 338)	Total (n = 1340)	DM (n = 317)	NDM (n = 41)	IGR (n = 105)	Normal (n = 109)	Total (n = 572)
β-Blocker	520 (82)	61 (74)	233 (82)	276 (82)	1090 (81)	225 (71)	29 (71)	85 (81)	85 (78)	424 (74)
ACEI/ARB	553 (87)	70 (84)	254 (90)	290 (86)	1167 (87)	251 (79)	32 (78)	88 (84)	86 (79)	457 (80)
Diuretics	153 (24)	18 (22)	55 (19)	40 (12)	266 (20)	109 (34)	6 (15)	12 (11)	17 (16)	144 (25)
Statins	563 (89)	75 (90)	252 (89)	312 (92)	1202 (90)	267 (84)	38 (93)	92 (88)	90 (87)	493 (86)
CCB	345 (54)	45 (54)	154 (54)	177 (52)	721 (54)	190 (60)	26 (63)	77 (73)	54 (50)	347 (61)
Nitrates	422 (66)	63 (76)	207 (73)	247 (73)	439 (70)	238 (75)	31 (76)	64 (61)	74 (68)	407 (71)
Aspirin	613 (96)	82 (99)	276 (98)	334 (99)	1305 (97)	282 (89)	41 (100)	96 (91)	107 (98)	526 (92)
Clopidogrel	538 (85)	80 (96)	254 (90)	317 (94)	1189 (89)	254 (80)	35 (85)	85 (81)	89 (82)	463 (81)
*DM drugs*										
Oral agents	364 (67)					170 (53)				
Insulin	147 (23)					73 (23)				
Combined	77 (12)					40 (13)				
No drugs/ Diet	48 (8)					34 (11)				

Data were number of patients (%)

ACEI, angiotensin converting enzyme inhibitor; ARB, angiotensin receptor blocker; CCB, calcium channel blocker; DM, diabetes mellitus; NDM, new diabetes mellitus; IGR, impaired glucose regulation

23% of diabetic patients in both groups were on insulin therapy, whereas oral hypoglycemic drugs were more commonly used in male diabetics. Besides, there was no difference in the proportion of male and female diabetics who were on combined treatment or on diet.

### Echocardiography findings

Echocardiography findings were shown in Table 5. Left and right atrial end-systolic diameters, left ventricular end-diastolic diameter and the ejection fraction were significantly different in both diabetic males and females compared to their non-diabetic counterparts respectively (*p* < 0.05). Comparing echocardiography findings in males with IGR and those without diabetes, there was a significant difference only in left atrial dimension and ejection fraction (*p* < 0.05) whereas there is no significant difference in any echocardiography finding between females with IGR and those with no DM.

**Table 5:** Echocardiography findings

	Male(n = 1340)				Female(n = 572)			
	DM (n = 636)	NDM (n = 83)	IGR (n = 283)	Normal (n = 338)	DM (n = 317)	NDM (n = 41)	IGR (n = 105)	Normal (n = 109)
L.A	3.9 (3.6,4.3)	3.7 (3.3,4.1)	3.7 (3.5,4.1)	3.7 (3.4,4.0)	3.8 (3.5,4.2)	3.8 (3.4,4.0)	3.6 (3.4,3.9)	3.6 (3.3,4.1)
L.V	5.0 (4.7,5.5)	5.0 (4.7,5.4)	4.9 (4.6,5.3)	4.8 (4.5,5.2)	4.8 (4.5,5.3)	4.7 (4.5,5.1)	4.6 (4.3,4.8)	4.6 (4.4,5.0)
R.A	3.7 (3.5,4.0)	3.8 (3.5,4.0)	3.7 (3.5,4.0)	3.7 (3.4,3.9)	3.6 (3.3,3.9)	3.4 (3.2,3.8)	3.4 (3.2,3.6)	3.5 (3.3,3.7)
R.V	3.6 (3.3,3.8)	3.6 (3.3,3.9)	3.6 (3.3,3.8)	3.6 (3.3,3.8)	3.4 (3.2,3.7)	3.3 (3.0,3.5)	3.3 (3.1,3.6)	3.4 (3.2,3.6)
Ao	3.4 (3.2,3.7)	3.5 (3.2,3.6)	3.4 (3.1,3.7)	3.4 (3.2,3.6)	3.3 (3.1,3.6)	3.3 (3.1,3.5)	3.4 (3.0,3.6)	3.3 (3.1,3.6)
EF	60	60	61	64	62	64	66	66
	(50,67)	(47,66)	(55,68)	(56,70)	(50,69)	(59,70)	(62,70)	(60,70)
IVS	1	1	1	1	1	1	0.9	0.9

Data are median (lower–upper quartiles)

LA, left atrium; RA, right atrium; LV, left ventricle; RV, right ventricle; Ao, aortic root; IVS, interventricular septum; EF, ejection fraction; DM. diabetes mellitus; NDM, new diabetes mellitus; IGR, impaired glucose regulation

### Lipid profile and blood glucose analysis

Table 6 summarized the hematological analysis. There were no significant differences (*p* > 0.05) in the total cholesterol and LDL-C levels between patients with IGR and diabetes compared to non-diabetic patients. On the contrary, HDL-C levels were significantly higher in normal patients (*p* < 0.05) compared to male and female diabetics, as well as female IGR patients. Besides triglyceride levels was significantly higher only in diabetic men relative to normal ones (*p* < 0.05) while no similar results were encountered in the remaining groups.

**Table 6:** Lipid profile and blood glucose analysis of patients

	Male (n = 1340)				Female (n = 572)			
	DM (n = 636)	NDM (n = 83)	IGR (n = 283)	Normal (n = 338)	DM (n = 317)	NDM (n = 41)	IGR (n = 105)	Normal (n = 109)
Lipid profile								
TC	3.81 (3.28,4.55)	3.92 (3.50,5.08)	4.07 (3.41,4.71)	3.99 (3.38,4.61)	4.05 (3.47,4.76)	4.31 (3.67,5.02)	4.17 (3.58,4.83)	4.46 (3.70,4.96)
HDL-C	1.04 (0.88,1.24)	1.02 (0.90,1.24)	1.10 (0.94,1.28)	1.12 (0.97,1.30)	1.16 (0.95,1.39)	1.27 (1.08,1.54)	1.23 (1.07,1.45)	1.34 (1.14,1.54)
LDL-C	1.96 (1.54,2.51)	2.22 (1.77,3.05)	2.20 (1.64,2.66)	2.13 (1.62,2.63)	2.11 (1.61,2.75)	2.41 (1.61,2.82)	2.03 (1.60,2.52)	2.31 (1.71,2.71)
TG	1.52 (1.10,2.20)	1.48 (1.12,2.31)	1.39 (1.04,2.04)	1.35 (0.98,1.93)	1.44 (1.06,2.02)	1.48 (1.15,2.04)	1.54 (1.10,2.01)	1.39 (1.01,1.84)
Blood glucose								
RBG	10.4 (7.6,12.7)	9.7 (8.1,13.1)	6.8 (5.4,7.6)	6.3 (5.3,7.3)	13.2 (9.6,15.6)	10.1 (8.0,11.5)	7.4 (6.2,10.2)	6.7 (6.4,7.3)
FBG	6.7 (5.3,7.9)	5.7 (4.9,6.5)	4.9 (4.4,5.4)	4.7 (4.0,5.0)	7 (6.1,9.6)	5.6 (5.6,6.6)	4.9 (4.8,5.0)	4.8 (4.5,5.1)
OGTT- 0Hr	5.7 (4.8,6.6)	5.1 (4.8,5.6)	4.4 (4.0,5.2)	4.4 (4.1,4.8)	6.4 (5.1,9.1)	5.4 (4.9,5.9)	4.6 (4.4,4.9)	4.4 (4.1,4.5)
OGTT—2Hr	14.2 (12.0,15.3)	13.2 (12.5,15.0)	9.1 (8.5,9.7)	6.0 (5.3,7.0)	13.6 (12.1,15.8)	12.3 (11.6,13.6)	8.3 (7.8,8.6)	6.1 (5.6,6.7)
HbA1C	6.6 (6.2,7.6)	6 (5.9,6.4)	5.7 (5.3,6.4)	5.6 (5.4,5.8)	7.2 (6.6,8.5)	6.1 (6.1,6.2)	6.1 (5.9,6.2)	5.6 (5.3,5.9)

Data were median (lower–upper quartiles)

TC, total cholesterol; HDL-C, high density lipoprotein-cholesterol; LDL-C, low density lipoprotein-cholesterol; TG, triglyceride; RBG, random blood glucose; FBG, fasting blood glucose; DM, diabetes mellitus; NDM, new diabetes mellitus; IGR, impaired glucose regulation

Concerning the blood glucose analysis, all the parameters were significantly higher in the diabetic than the normal groups in men as well as women but no similar trend was noted when comparing IGR patients to those with no DM.

### Glucometabolic status analysis

The glucometabolic states were categorized as shown in Table 7. In this population, 24% (n = 953) of the individuals were known to have type 2 diabetes mellitus, amongst which 23% were male (n = 636) and 27% (n = 317) were female. 959 patients (male, n = 704; female, n = 255) underwent OGTT in order to assess their glucometabolic state. The test showed that 83 male patients (12%) and 41 female patients (16%) had type 2 diabetes (*p* > 0.05). In addition, another 40% (n = 283) of men and 41% (n = 105) of female had IGR (which refers to the presence of either IFG or IGT) (*p* > 0.05). Thus, among the classified group, only 19% (n = 109) women and 25% (n = 338) men showed the normal glucose regulation, implying a higher prevalence of abnormal glucose regulation in females (*p* < 0.01). The prevalence of DM was higher in females than male (*p* < 0.01), while prevalence of IGR was not significantly different (*p* = 0.17) in the 2 groups. The odd ratio (OR) among the classified group showed that women were significantly more prone to have diabetes mellitus or IGT than men,with an OR of 1.44 and 1.43 respectively.

**Table 7:** Glucose regulation in men and women in the study population

Population	Glucometabolic status					Total	*P* value
	DM	NDM	IGT	IFG	Normal		
Women	317	41	97	8	109	572	0.003
Men	636	83	274	9	338	1340	
Total	953	124	371	17	447	1912	

DM, diabetes mellitus; NDM, new diabetes mellitus; IGT, impaired glucose tolerance; IFG, impaired fasting glycemia

Pearson Chi-Square Test is used and a value of *p* < 0.05 is considered significant

## Discussion

In this study, we investigated the prevalence of abnormal glucose regulation in men and women in Chinese CAD patients.We found that there was a high prevalence of abnormal glucose metabolism in patients with CAD. The prevalence of diabetes mellitus and IGT was more common in women than men.

One study reported that 9.7% of the adult population (10.6% men and 8.8% women) were suffering from DM and 15.5% had prediabetes (16.1% men and 14.9% women) in China [3], among general population of China. We found that 75% men and 81% women with CAD had abnormal glucose regulation, which is consistent with the China Heart Survey [27], which revealed that about 80% of CAD patients had AGR.

Several studies indicated a greater relative risk of CAD with diabetes [16, 28] and hyperglycaemia [29–31] among women than men, though absolute risk of CAD in male is higher regardless of diabetes. In accordance with the International Diabetes Federation, type 2 diabetes mellitus was more common in males than females in general population, however, there will be a little gender difference in 2010 and 2030. In 2010, it was expected that 1.1 million more women than men would suffer from diabetes (143 million women vs 142 million men) and this difference is anticipated to be almost six million by 2030 (222 million vs 216 million). However, in Chinese population, prevalence of type 2 diabetes and IGT is still more common in men. In this study, we demonstrated there was a female predominance in AGR with respect to CAD patients. According to our data, 27% of women (n = 317) were known diabetic patients compared to 23% male (n = 636), and a further 16% women and 12% men were newly diagnosed diabetics after OGTT. The proportion of patients having type 2 diabetes at the beginning of the survey was lower as compared to that of China Heart Survey (32.8%) [27] and Euro Heart Survey (31%) [32], which may be due to the fact that many patients had no screening for diabetes before admission. Among the classified patients, no more than 19% (n = 109) women and 25% (n = 338) men were with normal glucose regulation. Our finding was in agreement with a previous study in Europe [33], which showed similar percentage of women with normal glucose metabolism while that of men corresponded to 27%. Besides, prevalence of IGR was only slightly elevated in females than males (41% vs 40%), which was 39% vs 35% in previous study.

As previously reported [34–36], there is a gender disparity in management of CAD in men and women. The main reasons for this difference included gender bias, clinical inertia, financial grounds and other socio-economic issues. In our study, we found that a majority of patients were administered with the evidence-based drug regimen (aspirin, beta-blockers, statins and ACE inhibitors or angiotensin receptor blockers [ARB]) as recommended by guidelines for the treatment of patients with established CAD [37]. Slightly larger proportion of male patients were receiving β-blockers, statins, ACEI/ARB and antithrombotic drugs than their female counterparts. In China, the increasing number of patients on appropriate treatment for CAD is due to recent advances in the medical field and a higher level of public awareness about this disease. In addition, it is noteworthy that more patients were on double anti-platelet therapy, nitrates and CCBs than Euro Heart Survey [33]. Myocardial infarction was the main diagnosis at admission in Europe, however, it was unstable angina in our study. Besides, there was a higher percentage of females than males on diuretics and nitrates, which can be explained by higher prevalence of heart failure among the former. Unstable angina pectoris was the most common diagnosis among our study population, in line with China Heart Survey [27], There was a predominance in female patients and myocardial infarction presented more commonly in males [38]. Moreover, hypertension and heart failure were more prevalent in females, which may further contribute to increase relative risk of mortality in diabetic females when compared to males with CAD. Studies showed an inverse relationship between high-density lipoprotein cholesterol (HDL-C) levels and the risk of coronary heart disease [39, 40]. Walden et al. further explained the gender difference by stating that HDL cholesterol levels are lower in diabetic women than in diabetic men [41]. In our study, the HDL was higher in women diabetics. Due to the lipid-lowering treatment, the comparison between groups was inaccurate and unreliable.

## Conclusion

In conclusion, in this study, we found that glucometabolic abnormalities were very common in patients with CAD, and they were more prevalent in women than in men.

## Data Availability

All data generated or analyzed during this study are included in this published article.

## Abbreviations

CAD: Coronary artery disease
OR: Odd ratio
IGT: Impaired glucose tolerance
ACS: Acute coronary syndrome
IGR: Impaired glucose regulation
IFG: Impaired fasting glycemia
MI: Myocardial infarction
STEMI: ST-segment elevation MI
NSTEMI: Non-ST-segment elevation
RBG: Random blood glucose
FBG: Fasting blood glucose
ARB: Angiotensin receptor blockers
HDL-C: High-density lipoprotein cholesterol
